# Supplementary material for: Pseudocapacitive Behavior of Blade-Coated Mo1.33CTx i-MXene Electrodes in Aqueous Electrolytes
Source: Nanomaterials (Basel). 2025 Oct 19;15(20):1593. doi: 10.3390/nano15201593 (PMC12566924; doi:10.3390/nano15201593)
Supplement: Supplementary file 1 [file nanomaterials-15-01593-s001.zip › nanomaterials-3897792-supplementary.pdf]

Supporting Information

Pseudocapacitive Behavior of Blade-Coated $Mo_{1.33}CT_x$ i-MXene Electrodes in Aqueous Electrolytes

Alexey Tsyganov[a]*, Olga Grapenko[b], Evgeniy Korotaev[c], Alexander Shindrov[d], Andrei Alferov[a], Alexander Gorokhovsky[a] and Nikolay Gorshkov[a]*

[a] Department of Chemistry and Technology of Materials, Yuri Gagarin State Technical University of Saratov, 77 Polytecnicheskaya Street, 410054 Saratov, Russia.

[b] Research Institute of Physics, Southern Federal University, 194 Stachki Avenue,344011 Rostov-on-Don, Russia.

[c] Nikolaev Institute of Inorganic Chemistry, Siberian Branch, Russian Academy of Sciences, 630090 Novosibirsk, Russia

[d] Institute of Solid State Chemistry and Mechanochemistry, Siberian Branch of the Russian Academy of Sciences, 18 Kutateladze, 630128 Novosibirsk, Russia

Correspondence: Alexey Tsyganov and Nikolay Gorshkov

Email: tsyganov.a.93@mail.ru (A. Tsyganov); gorshkov.sstu@gmail.com (N. Gorshkov).

Table S1. Summary of global atomic percentages (at.%) of $Mo_{1.33}CT_x$ samples obtained from their XPS survey spectra.

MXene film	Mo, at.%	Y, at.%	C, at.%	O, at.%	Cl, at.%	F, at.%
$Mo_{1.33}CT_x$	17.8	1.0	54.0	23.4	2.3	1.5

Table S2. XPS peak fitting results for $Mo_{1.33}CT_x$ MXene.

Region	BE[eV][a]	FWHM[eV]	Fraction	Assigned to	Ref.
$Mo\ 3d_{5/2}(3d_{3/2})$	229.5 (232.7)	0.7(0.7)	0.38	C-Mo-T_z	[1]
	230.0 (233.1)	1.3(1.3)	0.20	Mo^{5+}	
	232.3 (235.4)	2.2(2.2)	0.42	Mo^{6+}	
C 1s	282.8	1	0.10	C-Mo-T_z	[2]
	284.8	1.7	0.75	C-C	
	286.6	1.4	0.08	C-O	
	288.6	1.8	0.07	COO	
O 1s	530.6	1.4	0.48	MoO_x	[3]
	531.5	1.5	0.24	C-Mo-O_x	
	532.6	1.4	0.20	C-Mo-$(OH)_x$	
	533.7	1.5	0.08	C-Mo-H_2O_{ads}	
F 1s	685.8	2.9	0.89	C-Mo-F_x	[4,5]
	689.0	2.7	0.11	AlF_3/AlO_xF_y	
$Y\ 3d_{5/2}(3d_{3/2})$	158.5 (160.5)	1.9(1.8)	0.17	C-Y-T_z	[5, 6]
	160.1 (162.2)	1.8(1.8)	0.83	Y-O	
$Cl\ 2p_{3/2}(2p_{1/2})$	198.7(200.4)	1.5 (1.3)	1	C-Mo-Cl_x	[7]

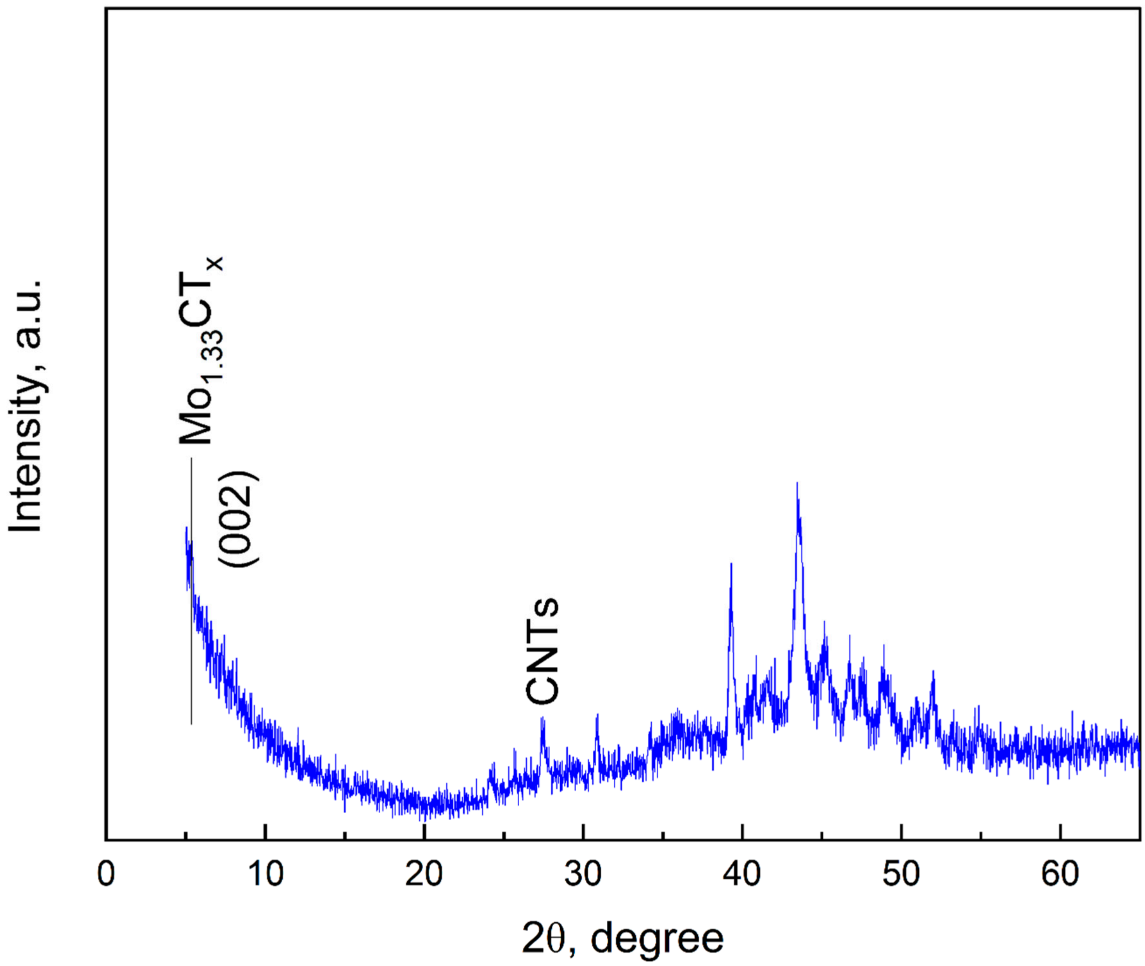

Figure S1. XRD pattern of the $Mo_{1.33}CT_x$-CNTs-PVDF coating on stainless steel foil.

Fig. S1 shows the XRD pattern of the $Mo_{1.33}CT_x$-CNTs-PVDF electrode coating on a stainless steel foil substrate. The (002) diffraction peak characteristic of $Mo_{1.33}CT_x$ is observed at $2\theta = 5.4°$. In the pure $Mo_{1.33}CT_x$ film (Fig. 1a), this peak was located at $2\theta = 8°$. This peak shift indicates the successful intercalation of carbon nanotubes between the $Mo_{1.33}CT_x$ nanosheets, resulting in an increase in the interlayer spacing. Furthermore, the low intensity of this diffraction peak suggests a disruption of the ordered stacking of the nanosheets. Thus, the incorporation of carbon nanotubes can result in a more chaotic orientation of the MXene nanosheets within the electrode structure.

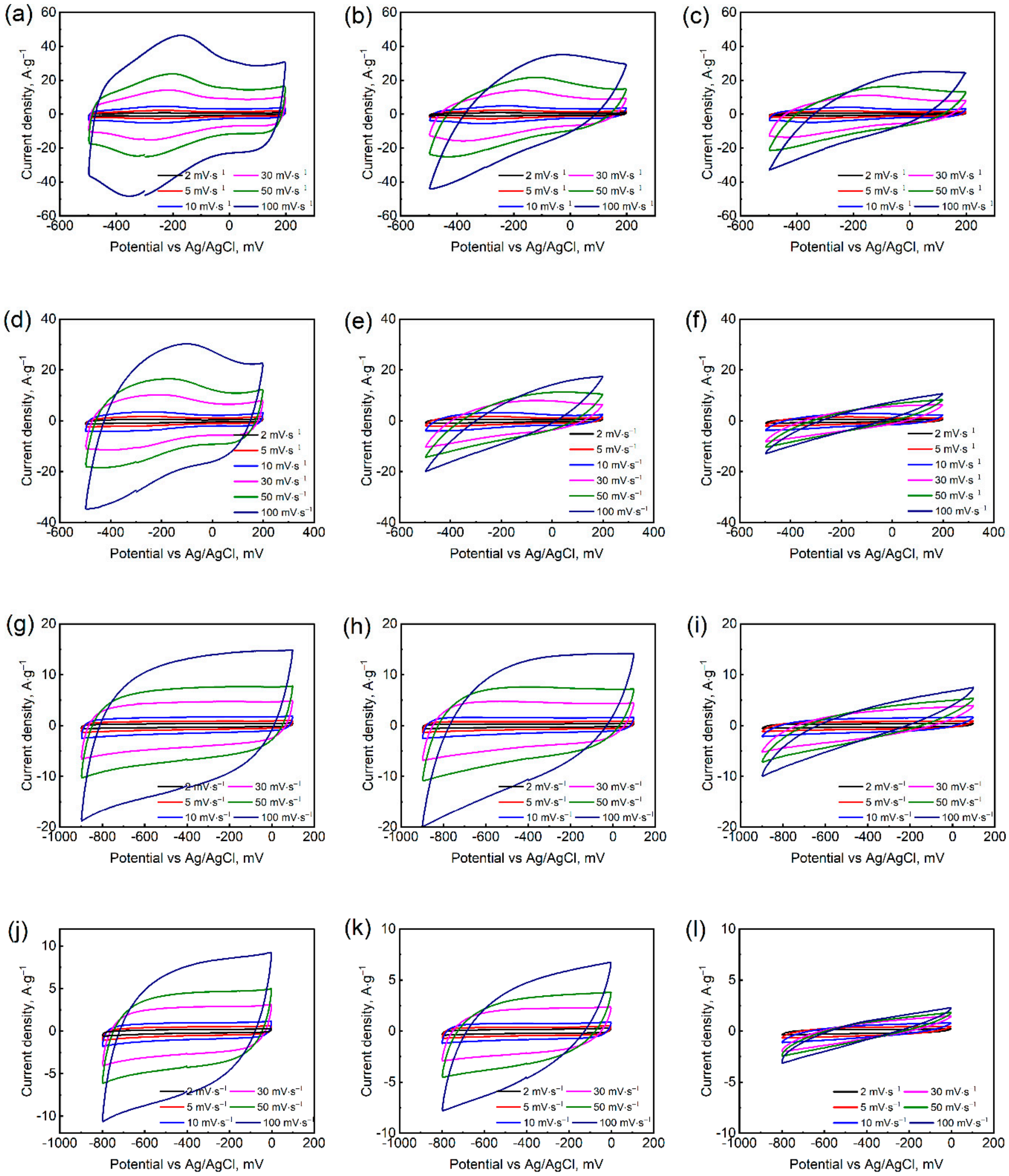

Figure S2. CV curves of $Mo_{1.33}CT_x$ MXene electrodes with different mass loadings. **(a)** 1.6 mg·cm^{-2} in H_2SO_4, **(b)** 3.5 mg·cm^{-2} in H_2SO_4, **(c)** 5.3 mg·cm^{-2} in H_2SO_4, **(d)** 1.6 mg·cm^{-2} in H_3PO_4, **(e)** 3.5 mg·cm^{-2} in H_3PO_4, **(f)** 5.3 mg·cm^{-2} in H_3PO_4, **(g)** 1.6 mg·cm^{-2} in LiCl, **(h)** 3.5 mg·cm^{-2} in LiCl, **(i)** 5.3 mg·cm^{-2} in LiCl, **(j)** 1.6 mg·cm^{-2} in KCl, **(k)** 3.5 mg·cm^{-2} in KCl, **(l)** 5.3 mg·cm^{-2} in KCl.

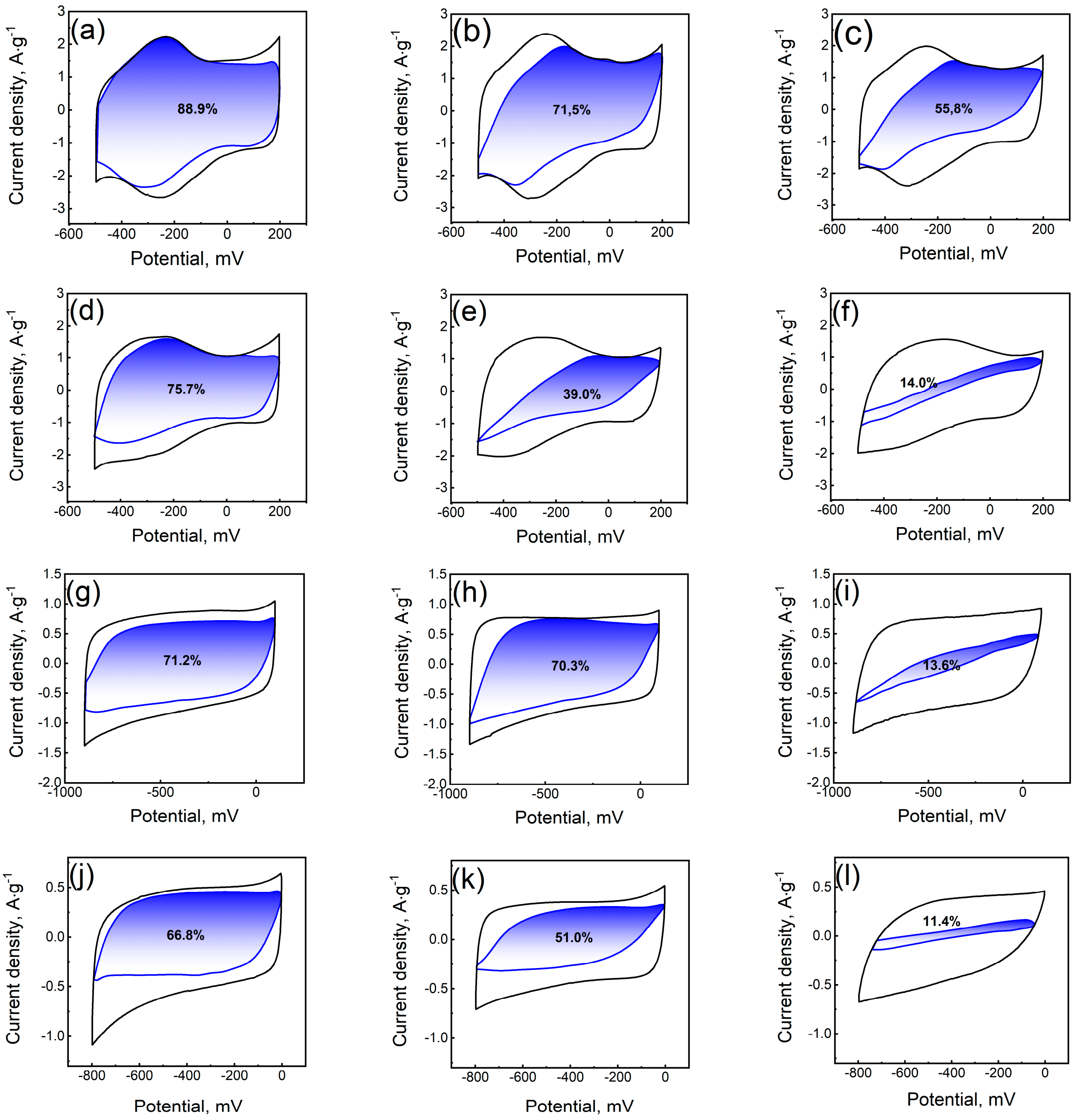

Figure S3. Surface-capacitive contribution to $Mo_{1.33}CT_x$ electrodes with different mass loadings at a scan rate of 5 mV·s^{-1}**.** **(a)** 1.6 mg·cm^{-2} in H_2SO_4, **(b)** 3.5 mg·cm^{-2} in H_2SO_4, **(c)** 5.3 mg·cm^{-2} in H_2SO_4, **(d)** 1.6 mg·cm^{-2} in H_3PO_4, **(e)** 3.5 mg·cm^{-2} in H_3PO_4, **(f)** 5.3 mg·cm^{-2} in H_3PO_4, **(g)** 1.6 mg·cm^{-2} in LiCl, **(h)** 3.5 mg·cm^{-2} in LiCl, **(i)** 5.3 mg·cm^{-2} in LiCl, **(j)** 1.6 mg·cm^{-2} in KCl, **(k)** 3.5 mg·cm^{-2} in KCl, **(l)** 5.3 mg·cm^{-2} in KCl.

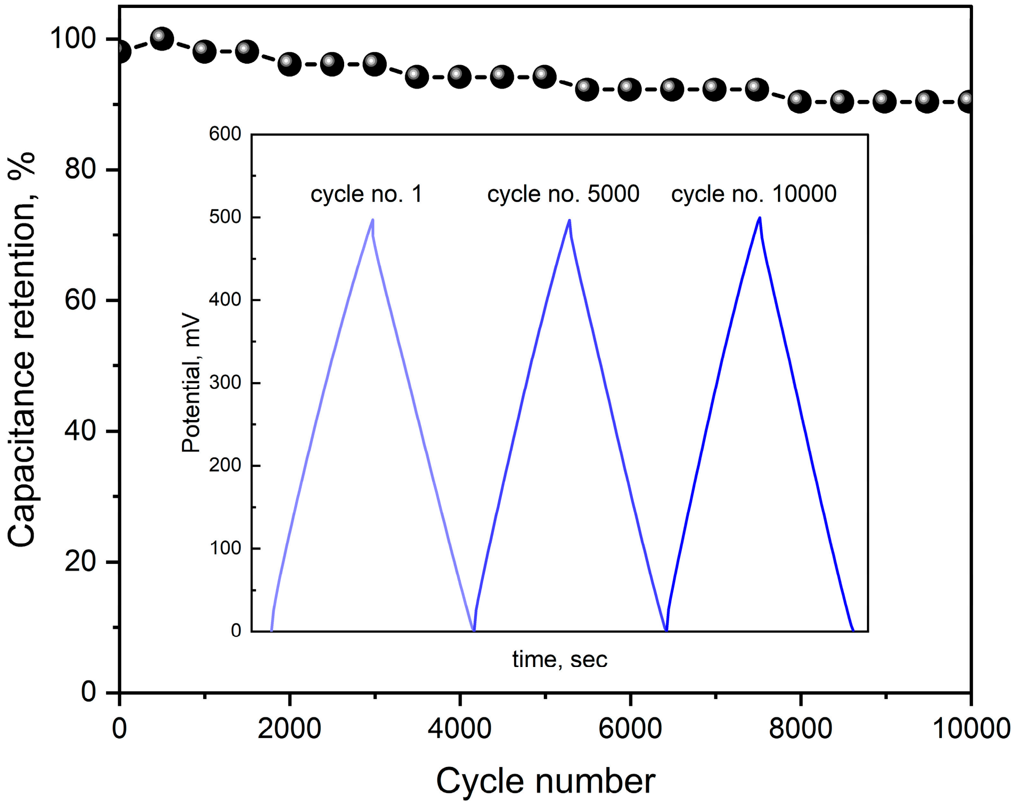

Figure S4. The cycling stability of $Mo_{1.33}CT_x$ electrodes in H_2SO_4 electrolyte at a current density of 10 $A{\cdot}g^{-1}$.

References

[1] J. Halim, S. Kota, M.R. Lukatskaya, M. Naguib, M.Q. Zhao, E.J. Moon, J. Pitock, J. Nanda, S.J. May, Y. Gogotsi, M.W. Barsoum, Synthesis and Characterization of 2D Molybdenum Carbide (MXene), Adv. Funct. Mater. 26 (2016) 3118–3127.

[2] H. Lind, J. Halim, S.I. Simak, J. Rosen, Investigation of vacancy-ordered M o1.33 C MXene from first principles and x-ray photoelectron spectroscopy, Phys. Rev. Mater. 1 (2017) 044002.

[3] V. Natu, M. Benchakar, C. Canaff, A. Habrioux, S. Célérier, M.W. Barsoum, A critical analysis of the X-ray photoelectron spectra of Ti3C2Tz MXenes, Matter 4 (2021) 1224–1251.

[4] J. Yang, R. Liu, N. Jia, K. Wu, X. Fu, Q. Wang, W. Cui, Novel W-based in-plane chemically ordered (W2/3R1/3)2AlC (R = Gd, Tb, Dy, Ho, Er, Tm and Lu) MAX phases and their 2D W1.33C MXene derivatives, Carbon N. Y. 183 (2021) 76–83.

[5] S. Sun, J. Yang, X. Chen, W. Cui, J. Huang, T. Yang, Z. Zhang, Q. Wang, A novel two-dimensional rare-earth carbide synthesized by selective etching Al-C slab from nanolaminated YAl3C3, Scr. Mater. 181 (2020) 10–14.

[6] D.L. Druffel, M.G. Lanetti, J.D. Sundberg, J.T. Pawlik, M.S. Stark, C.L. Donley, L.M. McRae, K.M. Scott, S.C. Warren, Synthesis and Electronic Structure of a 3D Crystalline Stack of MXene-Like Sheets, Chem. Mater. 31 (2019) 9788–9796.

[7] T. Bashir, S.A. Ismail, J. Wang, W. Zhu, J. Zhao, L. Gao, MXene terminating groups O, –F or –OH, –F or O, –OH, –F, or O, –OH, –Cl?, J. Energy Chem. 76 (2023) 90–104.
